# Comparison of malignancy‐prediction efficiency between contrast and non‐contract CT‐based radiomics features in gastrointestinal stromal tumors: A multicenter study

**DOI:** 10.1002/ctm2.91

**Published:** 2020-07-07

**Authors:** Qing‐Wei Zhang, Xiao‐Xuan Zhou, Ran‐Ying Zhang, Shuang‐Li Chen, Qiang Liu, Jian Wang, Yan Zhang, Jiang Lin, Jian‐Rong Xu, Yun‐Jie Gao, Zhi‐Zheng Ge

**Affiliations:** ^1^ Division of Gastroenterology and Hepatology Key Laboratory of Gastroenterology and Hepatology Ministry of Health, Renji Hospital, School of Medicine, Shanghai Jiao Tong University, Shanghai Institute of Digestive Disease Shanghai China; ^2^ Department of Radiology Sir Run Run Shaw Hospital (SRRSH), School of Medicine, Zhejiang University Hangzhou China; ^3^ Department of Radiology Zhongshan Hospital, Fudan University, and Shanghai Institute of Medical Imaging Fenglin Road 180 Shanghai 200032 China; ^4^ Department of Radiology The First Affiliated Hospital of Wenzhou Medical University Wenzhou China; ^5^ Department of Pathology Renji Hospital School of Medicine Shanghai Jiao Tong University Shanghai China; ^6^ Department of radiology Tongde Hospital of Zhejiang Province Hangzhou China; ^7^ Department of Radiology Renji Hospital School of Medicine Shanghai Jiao Tong University Shanghai China

**Keywords:** gastrointestinal stromal tumor, malignant potential, prediction, radiomics signature

## Abstract

This work seeks the development and validation of radiomics signatures from nonenhanced computed tomography (CT, NE‐RS) to preoperatively predict the malignancy degree of gastrointestinal stromal tumors (GISTs) and the comparison of these signatures with those from contrast‐enhanced CT. A dataset for 370 GIST patients was collected from four centers. This dataset was divided into cohorts for training, as well as internal and external validation. The minimum‐redundancy maximum‐relevance algorithm and the least absolute shrinkage and selection operator (LASSO) algorithm were used to filter unstable features. (a) NE‐RS and radiomics signature from contrast‐enhanced CT (CE‐RS) were built and compared for the prediction of malignancy potential of GIST based on the area under the receiver operating characteristic curve (AUC). (b) The radiomics model was also developed with both the tumor size and NE‐RS. The AUC values were comparable between NE‐RS and CE‐RS in the training (.965 vs .936; *P* = .251), internal validation (.967 vs .960; *P* = .801), and external validation (.941 vs .899; *P* = .173) cohorts in diagnosis of high malignancy potential of GISTs. We next focused on the NE‐RS. With 0.185 selected as the cutoff of NE‐RS for diagnosis of the malignancy potential of GISTs, accuracy, sensitivity, and specificity for diagnosis high‐malignancy potential GIST was 90.0%, 88.2%, and 92.3%, respectively, in the training cohort. For the internal validation set, the corresponding metrics are 89.1%, 94.9%, and 80.0%, respectively. The corresponding metrics for the external cohort are 84.6%, 76.1%, and 91.0%, respectively. Compared with only NE‐RS, the radiomics model increased the sensitivity in the diagnosis of GIST with high‐malignancy potential by 5.9% (*P* = .025), 2.5% (*P* = .317), 10.5% (*P* = .008) for the training set, internal validation set, and external validation set, respectively. The NE‐RS had comparable prediction efficiency in the diagnosis of high‐risk GISTs to CE‐RS. The NE‐RS and radiomics model both had excellent accuracy in predicting malignancy potential of GISTs.

## Graphical abstract

A dataset for 370 GIST patients was collected from four centers and was divided into cohorts for training, as well as internal and external validation. The minimum‐redundancy maximum‐relevance (mRMR) algorithm, and the least absolute shrinkage and selection operator (LASSO) algorithm were used to filter unstable features. The area under the curve (AUC) values were comparable between radiomics signatures from non‐enhanced CT (NE‐RS) and radiomics signatures from enhanced CT (CE‐RS) in the three cohorts in diagnosis of high malignancy potential of GISTs. With 0.185 selected as the cut‐off of NE‐RS for diagnosis of the malignancy potential of GISTs, accuracy for diagnosis high‐malignancy potential GIST was 89%, 85% for the internal validation and external validation cohort. Compared with only NE‐RS, the radiomics model with combination of radiomic signature and tumor size, had increased accuracy of 91%, 89% for the internal validation and external validation cohort. The NE‐RS and radiomics model both had excellent accuracy in predicting malignancy potential of GISTs.

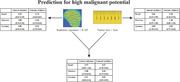

**Abbreviations**
2D: two‐dimensional
AUC: area under the receiver operating characteristic curve
CE‐CT: contrast‐enhanced computed tomography
CE‐RS: radiomics signature from contrast‐enhanced CT
CT: computed tomography
GIST: gastrointestinal stromal tumors
ROI: region of interest

Gastrointestinal stromal tumors (GISTs) are among the most common subepithelial tumors in the digestive system. Different treatment strategies have been applied to treat GIST, including close follow‐up, submucosal endoscopic dissection, endoscopic full‐thickness resection, and surgery.^1^, ^2^, ^3^ Although risk classifications were developed and validated in clinical practice,^4^, ^5^ all of the proposed risk classifications were based on histological examination and were applied postoperatively. On the other hand, endoscopic performance, clinical symptoms, and findings from computed tomography (CT)^6^ are useful and typically combined in the GIST preoperative risk stratification for subsequent treatment decision. However, the assessments mentioned above are subjective based on the experience of observers.

Radiomics represents an emerging tool for extracting numerous numerical features from medical images and has gained popularity in cancer diagnosis.^7^, ^8^, ^9^ Previous studies have identified radiomics features from contrast‐enhanced CT (CE‐CT) as a superior tool for predicting the malignancy potential of GIST compared with clinical factors.^10^, ^11^ However, whether radiomics features extracted from nonenhanced CT are useful for preoperative GIST malignancy assessment is still unknown. Therefore, we assessed whether radiomics signature from nonenhanced CT (NE‐RS) is useful in predicting potentially malignant GIST compared to those from contrast‐enhanced CT (CE‐RS).

We recruited 370 patients with GIST from four hospitals according to the following criteria: (a) patients who had surgeries or endoscopic resections; (b) both conventional CT and arterial phase CE‐CT examinations were done within a 15‐day period before treatment; (c) GIST diagnosis was carried out with histological and immunohistochemical tests; and (d) all reported clinical and pathological variables were available. Patients who received or were revived with imatinib preoperatively or those with multiple GIST detections were excluded. Ethical approvals were obtained for all four collaborating hospitals. The training cohort consisted of patients diagnosed consecutively between January 2011 and December 2016 in Renji Hospital. The internal validation cohort contained patients from January 2017 to June 2019 in Renji Hospital. The external validation cohort included patients from three hospitals diagnosed between January 2017 and June 2019. We collected clinical and pathological variables including patient gender, age, tumor location, size, and mitotic count. GISTs were classified according to the National Institutes of Health‐modified criteria into two groups, GISTs with low potential of malignancy and GISTs with high potential of malignancy, based on pathological tumor size, tumor location, and mitotic count.^4^ Low‐malignancy‐potential category consisted of GISTs with very low or low risk. The high‐malignancy‐potential category included GISTs with intermediate or high risk. As shown in Table 1, tumor location, tumor size, and mitotic count, but not age or sex, were significantly associated with high‐malignancy‐potential GIST status in the univariate analysis model.

**Table 1:** Clinical characteristics of patients in the training and validation cohort

	Training cohort			Internal validation cohort			External validation cohort		
	Low‐malignant	High‐malignant	*P*‐value	Low‐malignant	High‐malignant	*P*‐value	Low‐malignant	High‐malignant	*P*‐value
Age, mean ± SD (years)	61.43 ± 11.02	62.34 ± 13.04	.644	64.08 ± 14.21	61.64 ± 14.25	.506	60.22 ± 10.28	60.87 ± 10.81	.709
Sex			.798			.124			.965
Female	29 (44.62%)	35 (41.18%)		16 (64.00%)	16 (41.03%)		49 (55.06%)	38 (56.72%)	
Male	36 (55.38%)	50 (58.82%)		9 (36.00%)	23 (58.97%)		40 (44.94%)	29 (43.28%)	
Location			.002			.006			.038
Stomach	47 (72.31%)	40 (47.06%)		22 (88.00%)	20 (51.28%)		73 (82.02%)	44 (65.67%)	
Nonstomach	18 (27.69%)	45 (52.94%)		3 (12.00%)	19 (48.72%)		16 (17.98%)	23 (34.33%)	
Size (cm)			<.001			<.001			<.001
≤5	65 (100%)	27 (31.76%)		25 (100%)	7 (17.95%)		89 (100%)	28 (41.79%)	
>5	0 (0)	58 (68.24%)		0 (0)	32 (82.05%)		0 (0)	39 (58.21%)	
Mitotic count			<.001			<.001			<.001
≤5/50 HPF	65 (100%)	51 (60.00%)		25 (100%)	26 (66.67%)		96 (100%)	36 (53.73%)	
>5/50 HPF	0 (0)	34 (40.00%)		0 (0)	13 (33.33%)		0 (0)	31 (46.27%)	

Abbreviations: HPF, high‐power field; SD, standard deviation.

Figure 1A demonstrates our workflow. The ITK‐SNAP software was used to examine the noncontrast CT and CE‐CT images for each tumor, and hence delineate regions of interest (ROI) on the slice of the largest tumor area. We used the open‐source PyRadiomics package^12^ to extract 833 features from the ROI of each GIST. For feature selection and signature construction, we selected 404 high reproducible features with on intra‐ or interclass correlation coefficients exceeding 0.80.^13^ Among these features, only the top 20 were selected using the minimum‐redundancy maximum‐relevance algorithm.^14^ Signatures for the prediction of high‐malignancy GIST were built from the selected features using the least absolute shrinkage and selection operator (LASSO) algorithm.^15^ In the training cohort, 14 and 13 final radiomics features were used for NE‐RS and CE‐RS development, respectively. The detailed method for recruitment and development of radiomics signature is shown in the Supporting Information A1. Detailed NE‐RS and CE‐RS formula are shown in the Supporting Information A2.

**Figure 1:**
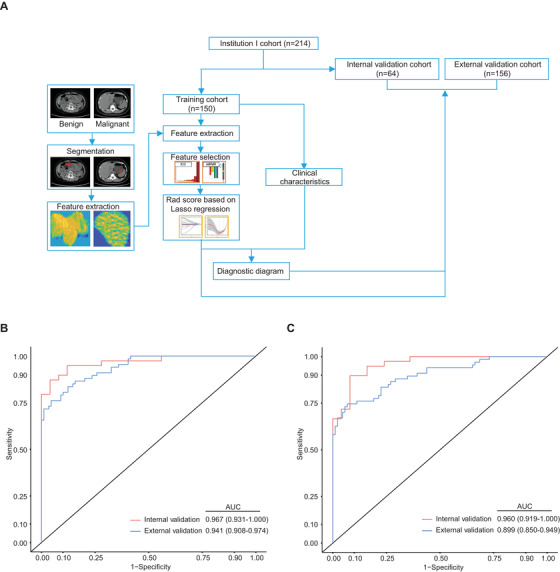
A, Schematic diagram of the proposed workflow. Based on the malignant potential profile, tumor area segmentation and feature extraction were performed. GIST patients were categorized into training, internal validation, and external validation cohorts. The training cohort data were subjected to further downstream processing and clinical tests. B, Receiver operating characteristic curves of the NE‐RS in predicting malignancy potential of GIST for the cohorts of internal validation and external validation. C, ROC curves of the CE‐RS in predicting malignancy potential of GIST for the cohorts of internal validation and external validation. Abbreviations: AUC, area under the receiver operating characteristic curve; CE‐RS, radiomics signature from contrast‐enhanced computed tomography; GIST, gastrointestinal stromal tumors; NE‐RS, radiomics signature from nonenhanced computed tomography.

Then, we analyzed and compared the NE‐RS and CE‐RS performance in GIST malignancy prediction using the area under the receiver operating characteristic curve (AUC) on the internal and external validation cohorts. As shown in Figure 1B, NE‐RS still had a high AUC of .967 (95% confidence interval [CI], .931‐1.00) on the internal validation cohort and an AUC of .941 (95% CI, .908‐.974) on the external validation cohort for GIST malignancy prediction. The CE‐RS had high AUC value of .960 (95% CI, .919‐1.00) and .899 (95% CI, .850‐.949) for predicting GIST malignancy potential on the internal and external validation cohorts, respectively. The DeLong test demonstrated no insignificant differences in the AUCs of the NE‐RS and the CE‐RS in the cohorts of internal validation (*P* = .801) and external validation (*P* = .173), which indicated similar predictive efficacy of malignancy potential in GIST. To our knowledge, two previous studies have developed the radiomics signatures extracted from CE‐CT for predicting GIST with high malignancy potential.^10^, ^11^ Chen et al^10^ used volumetric ROIs to develop signature with an AUC value of .867 in the training cohort and .847 in the external validation cohort.^10^ Wang et al^11^ used single‐slice ROIs to develop signature with an AUC value of .882 in the training cohort and .920 in the validation cohort.^11^ However, none of these studies explored radiomics signature from nonenhanced CT because it is a more commonly used screening tool for the detection and diagnosis of GIST. Our proposed NE‐RS had a higher AUC compared to the radiomics signatures developed in the other studies.^10^, ^11^ These studies used portal phase CE‐CT features to develop radiomics signature. On the other hand, clinical heterogeneity from different clinical setting, for example, different scanners, systems, or parameters, may explain it.

Because nonenhanced CT is more commonly used for preoperative GIST diagnosis and is similar to CE‐CT in prediction efficacy, we focused on the radiomics signature from nonenhanced CT. The optimal threshold of the radiomics signature for GIST malignancy diagnosis was set at the point of the ROC curve with the highest Youden index.^16^ Based on this thresholding method, NE‐RS cutoff value was set to .185. GIST samples with NE‐RS exceeding this value were identified as high‐malignancy‐potential GIST samples, whereas the other samples were identified as low‐risk samples. Performance metrics of precision, recall, average precision recall, accuracy, and the confusion matrix were computed for the radiomics signature using this threshold. As shown in Table 2, the precision, recall, accuracy, and average precision recall of the diagnosis of high‐malignancy‐potential GIST were .88 (95% CI, .74‐.96), .95 (95% CI, .81‐.99), .89 (95% CI, .80‐.96), and .98, respectively, for the internal validation cohort. The corresponding results for the external validation cohort are .76 (95% CI, .64‐.85), .86 (95% CI, .74‐.94), 0.85 (95% CI, .78‐.90), and .94, respectively.

**Table 2:** Performance evaluation of the radiomics models

	Internal validation cohort			External validation cohort		
	Radiomics signature	Size	Combination	Radiomics signature	Size	Combination
TP	37	32	38	51	39	58
TN	20	25	20	81	89	81
FN	2	7	1	16	28	9
FP	5	0	5	8	0	8
Recall	0.95 (0.81‐0.99)	0.82 (0.81‐0.99)	0.97 (0.85‐1.00)	0.76 (0.64‐0.85)	0.58 (0.46‐0.70)	0.87 (0.76‐0.93)
Precision	0.88 (0.74‐0.96)	1.00 (0.87‐1.00)	0.88 (0.74‐0.96)	0.86 (0.74‐0.94)	1.00 (0.89‐1.00)	0.88 (0.77‐0.94)
Accuracy	0.89 (0.80‐0.96)	0.89 (0.78‐0.95)	0.91 (0.80‐0.96)	0.85 (0.78‐0.90)	0.82 (0.75‐0.88)	0.89 (0.83‐0.93)
Average precision recall	0.98	0.95	0.98	0.94	0.89	0.94

Abbreviations: FN, false negative; FP, false positive; TN, true negative; TP, true positive;

Similar to other studies,^10^, ^11^ our multivariate logistic model recognized only tumor size and radiomics signature as potential risk factors for high‐malignancy GIST. According to the guidelines of the National Comprehensive Cancer Network (NCCN), GIST with a size exceeding 2 cm should be dealt with surgical resection, and tumors sized less than 2 cm could be monitored by endoscopy.^17^ As shown in Table 2, if a tumor size threshold of 5 cm is used for high‐malignancy GIST diagnosis, the internal and external validation cohorts will have misdiagnosis rates of 17.9% and 41.8%, respectively.

However, evaluation of malignancy only based on tumor size is not sufficient because tumors with mitotic count >5/50 HPF, regardless of tumor size, are considered aggressive with poor prognosis.^4^, ^18^, ^19^ If GIST samples with a tumor size exceeding 5 cm or NE‐RS exceeding the 0.185 threshold are identified as high‐malignant potential GIST samples (Table 2), the recall metric is increased by 2.5% (*P* = .317) and 10.5% (*P* = .008) for the cohorts of internal validation and external validation, respectively, in comparison to the case of GIST malignancy prediction with the NE‐RS thresholding. As shown in Table 2, using a tumor size of more than 5 cm led to 17.9‐41.8% of missed cases. Also, using a radiomics signature cutoff value of .185 led to 5.1‐23.9% of missed cases. When combining the tumor size and radiomics signature, 2.6‐13.4% of the cases were missed. Therefore, radiomics signature could help the diagnosis of GIST with high malignancy potential.

With the advancement in resection methods, multiple resection methods could be performed for the treatment of GIST, including submucosal endoscopic dissection, full‐thickness endoscopic resection, and surgery. However, the choice of the resection method is based on preoperative evaluation of tumors, including evaluation of tumor size, growth pattern, and malignancy. It is recommended that endoscopic resection with choice of submucosal endoscopic dissection or endoscopic full‐thickness resection based on the clinical decision should be performed for GIST sized <5 cm without high‐malignant potential and that GIST sized >5 cm or with high‐malignant potential should be resected by surgery.^1^, ^3^, ^20^, ^21^ Using our proposed radiomics model, 92.1% of GIST with high malignancy potential and 89.9% of GIST with low malignancy potential were correctly diagnosed and recommended for endoscopic resection.

In this study, radiomics features were extracted for a single two‐dimensional (2D) slice with the largest tumor area for each patient. No significant differences have been shown in the diagnostic efficacy between the texture analysis obtained from a single‐slice 2D slice versus a three‐dimensional volume.^22^ Segmentation of single‐slice 2D images could also save time compared with volumetric segmentation. In our study, a high AUC of .94 or more was found for the diagnosis of GIST with high‐malignancy potential in all three independent cohorts. Also, radiomics scores built from single‐slice 2D ROIs of CE‐CT achieved similar predictive accuracy compared to those from volumetric ROI of CE‐CT, in diagnosing GIST with high malignancy potential.^10^, ^11^ It confirmed that the single‐slice‐based 2D radiomics model might be feasible for the diagnosis of cancer‐related tumors.

The present work has some limitations. First of all, the dataset collection was done retrospectively. Thus, selective bias could not be eliminated. Nevertheless, patients were consecutively enrolled for bias reduction. Prospective studies are needed for the validation of our radiomics signature and model. Second, data heterogeneity bias resulted from CT parameter variations among the collaborating hospitals. Before the extraction of features, this bias was reduced through CT slice normalization and resampling. The *z*‐score method was applied to standardize the training features by their respective means and standard deviations. The AUC of NE‐RS did not vary among different hospitals. This demonstrated that our normalization method was reliable. Third, the predictive model did not account for gene mutations. Nevertheless, these variables could not be acquired by preoperative clinical tests and thus were not considered. Further investigations are needed to the explore the relationship of gene mutation and radiomics features.

In summary, we have developed and validated the NE‐RS with high performance in the diagnosis of GIST with high‐ and low‐malignancy potential and was comparable to the CE‐RS. Including tumour size and NE‐RS further increased the diagnostic accuracy in predicting GIST with high‐malignancy potential to make a preoperatively more precise clinical decision compared to only NE‐RS used.

## ETHICS APPROVAL AND CONSENT TO PARTICIPATE

The study protocol was approved by the Ethics Committee of the Renji Hospital, Zhongshan Hospital, Sir Run Run Shaw Hospital, and First Affiliated Hospital, Wenzhou Medical University. Informed consent was obtained from each patient before performing CE‐CT examination.

## AVAILABILITY OF DATA AND MATERIALS

The datasets generated and/or analyzed during the current study are not publicly available due personal information involved but are available from the corresponding author on reasonable request.

## CONFLICT OF INTEREST

The authors declare no conflict of interest.

## AUTHOR CONTRIBUTIONS

Q‐WZ, JL, J‐RX, Y‐JG, and Z‐ZG conceptualized and designed the study. Q‐WZ, X‐XZ, R‐YZ, S‐LC, YZ, QL, and JW helped with generation, collection, assembly, analysis, and/or interpretation of the data. Q‐WZ, X‐XZ, R‐YZ, and S‐LC drafted and revised the manuscript. Q‐WZ, JL, J‐RX, Y‐JG, and Z‐ZG approved the final version of the manuscript.

## FUNDING INFORMATION

This work was supported by grants from the National Natural Science Foundation of China (No. 81670505 and 81772519), the Shanghai Municipal Education Commission: Gaofeng Clinical Medicine grant support (No. DLY201501), the 3‐year action plan for Shin Kang of Shanghai (No. 16CR3113B), Cross Medical Research Fund of Translational Medicine, Shanghai Jiao Tong University (No. ZH2018ZDA06), Shanghai Municipal Key Clinical Specialty (No. shslczdzk05902), and Shanghai Municipal Commission of Health and Family Planning (No. 2018YQ29).

## Supporting information

Supporting Information
