# Supplementary material for: Comparison of malignancy‐prediction efficiency between contrast and non‐contract CT‐based radiomics features in gastrointestinal stromal tumors: A multicenter study
Source: Clin Transl Med. 2020 Jul 7;10(3):e291. doi: 10.1002/ctm2.91 (PMC7418807; doi:10.1002/ctm2.91)
Supplement: Supplementary file 1 — Supporting Information [file CTM2-10-e291-s001.docx]

**Supplementary A1 Method**

**Patient Data Collection**

Ethical approvals were obtained for all four collaborating hospitals. A set of 370 GIST patients were recruited in this patient study. All patents provided written informed consent. The patients who received or were revived imatinib preoperatively or those with multiple GIST detections were excluded. The inclusion criteria were set as: (1) patients who had surgeries or endoscopic resections; (2) Both conventional CT and arterial phase CE-CT examinations were done within a 15-day period before treatment; (3) GIST diagnosis was carried out with histological and immunohistochemical tests; (4) All reported clinical and pathological variables were available.

Enrolled patients were categorised into cohorts for development, as well as internal and external validation. The training cohort consisted of patients diagnosed consecutively between January 2011 and December 2016. The internal validation cohort contained patients undergoing surgeries from January 2017 to June 2019 from one hospital. The external validation cohort included patients from three hospitals diagnosed between January 2017 and June 2019.

**Clinical variables and primary outcomes**

Clinical variables and pathological characteristics were collected for each patient. These include the patient gender, age, tumour location, tumour size, and mitotic count. Tumour size was measured using CT images, and mitotic counted from pathological records. Mitotic count was classified as <=5/50 high-power field (HPF) and >5/50 HPF. GIST were classified according to the NIH modified criteria into two groups, GIST with low potential of malignancy and high potential of malignancy, based on pathological tumour size, tumour location, and mitotic count (1). Low-malignancy potential category consisted of GIST with very low or low risk. The high-malignancy potential category included GIST with intermediate or high risk.

**CT examination**

CT examination was performed for each patient within 14 days before treatment. At all four centres, patients underwent a similar scan setup but with different systems and parameters. All patients fasted overnight before the scan. Patients drank 600–1000 ml warm water or received 6 g of effervescent granules to distend the stomach before the CT scan. Twenty (20) mg of scopolamine or amidoamine was administered intramuscularly to reduce gastrointestinal peristalsis 15–20 min before the computed tomography (CT) scan. For small bowel scan, all patients fasted overnight and drank 2L polyethene glycol (PEG) or 1-2 L mannitol on the day of the exam. For large bowel scan, all patients fasted 4-6 hours before the examination on the same day.

CT scans spanning the entire stomach or bowel regions were obtained during a breath-hold with the patient in the supine position. The CT image acquisition parameters at all four centres are shown in Table S1.

The diagnosis was performed with a standardised dynamic window adjustment procedure, on a window-adjustable Picture Archiving and Communication System work station. A narrow window was used to demonstrate the primary gastrointestinal stromal tumours (GIST). Images in axial, coronal, and sagittal planes were observed simultaneously to facilitate the detection and location of the primary GIST.

**Radiomics features extraction**

Figure 1A demonstrates our workflow. Images of non-contrasted CT and CE-CT modalities were collected from picture archiving and communication system (PACS) files of each patient. Regions of interest (ROI) of these images were manually segmented by radiologists using the ITK-SNAP software (version 3.6.0). All CT slices of each patient were examined. Then, for each lesion, the slice of the largest tumour area was selected from the non-contrasted CT and CE-CT images. After that, two-dimensional (2D) ROIs were used to delineate the tumour region on those selected slices.

Before extracting the features, all arterial phase or non-enhanced CT images were normalised (the image intensity was scaled to 0–500) and resampled at the same resolution (1×1×1mm) before feature extraction to avoid data heterogeneity bias., which may occur when different scanners may produce different images for the same person. This method was used to avoid such bias.

After that, image smoothing and differencing were performed prior to feature extraction. To avoid multi-dimensional convolution, separable filters were used. The convolution was carried out with a low- (L) / or high (H) -pass “Coiflet 1” wavelet filter along the x- or y-direction through the application of different weights to the wavelet-domain band-pass and sub-bands (LHL, LHH, LLH, HLL, HHL and HLH) of the tumour region as compared to the low- or high-frequency sub-bands (LLL and HHH).

After filtering, a total of 833 features were extracted from the ROI within the input images, and its associated filtered results, including 18 features of the first-order statistics, 14 shape features, 22 GLCM features, 16 GLRLM features, 16 GLSZM features, 14 GLDM features, five NGTDM features, and 728 wavelet features. Image filtering and feature extraction were performed using PyRadiomics (2) in Python (3.7).

Radiomics features for all patients were standardised by the z-score method, based on training parameters.

**Radiomics feature selection and signature building process**

One radiologist with nine years of experience with CT images (reader 1) performed tumour segmentation on all image datasets. Three months after the initial segmentation, 40 training datasets were randomly selected and re-segmented by the same radiologist to assess intra-observer agreement for radiomics features. Then, these cases were segmented by another radiologist with five years of experience with CT images (reader 2) for assessing inter-observer agreement.

The feature selection and signature construction were performed in three steps. First, based on distinct segmentation groups, intra- or inter-class correlation coefficients (ICCs) were used to estimate the reproducibility of each feature (3). Stable features with both of intra- or inter-class ICC value greater than 0.8 were reserved.

Second, features were ranked using the mRMR algorithm (4) by calculating the mutual information (MI) between radiomics features and risk classification of GISTs and only 20 highest-ranking features in mRMR were reserved.

Third, the LASSO logistic regression model was used to build radiomics signature (5).

**Supplementary A2: Radiomics signatures calculation formula**

NE-RS= 0.551 + 0.837 × original_shape_Maximum2DDiameterColumn + 0.753 × wavelet.HLH_firstorder_Median – 0.191 × wavelet.LLL_ngtdm_Coarseness – 0.147 × wavelet.HLL_ngtdm_Contrast + 0.055 × wavelet.HHH_gldm_DependenceNonUniformityNormalized – 0.553 × wavelet.HLH_glcm_Id – 0.015 × wavelet.LLL_gldm_SmallDependenceLowGrayLevelEmphasis + 0.074 × wavelet.HLH_glszm_LargeAreaLowGrayLevelEmphasis + 0.054 × wavelet.LHH_firstorder_RootMeanSquared + 0.967 × wavelet.HLL_glszm_GrayLevelNonUniformity – 1.143 × wavelet.LLH_ngtdm_Coarseness + 0.173 × wavelet.HHL_glrlm_LongRunEmphasis – 0.363 × original_shape_SurfaceVolumeRatio – 0.590 × original_shape_Sphericity

CE-RS= 0.704 + 0.156 × wavelet.HLH_glcm_InverseVariance + 0.387 × original_shape_MajorAxisLength + 0.115 × original_glcm_Idn + 0.020 × wavelet.LLL_glcm_Correlation + 0.004 × wavelet.LLH_glszm_GrayLevelNonUniformity + 0.175 × wavelet.HHH_glrlm_RunVariance + 0.0005 × wavelet.HHH_gldm_DependenceNonUniformityNormalized + 0.139 × wavelet.HLL_glszm_SmallAreaEmphasis + 0.147 × wavelet.HLL_firstorder_RootMeanSquared + 0.390 × wavelet.HHL_glszm_ZoneEntropy – 0.423 × wavelet.HHH_ngtdm_Coarseness – 0.189 × wavelet.LHH_glcm_Id + 1.375 × original_shape_MinorAxisLength

**Table S1.** The CT parameters at the four centers

| Parameters | Center 1 | Center 2 | Center 3 | Center 4 |
| --- | --- | --- | --- | --- |
| CT version | Spectral CT (Discovery CT750 HD scanner, GE Healthcare, USA) | Spectral CT (Aquilion One TSX-301A, TOSHIBA, Japan) | Spectral CT (SOMATOM Sensation 16,  Siemens Forchheim Germany; Siemens Definition AS 40,  Siemens Healthcare Forchheim Germany; SOMATOM  Definition Flash, Siemens Healthcare Forchheim Germany;  LightSpeed VCT, GE Healthcare, Milwaukee, WI) | Spectral CT (Discovery CT750 HD scanner, GE Healthcare, USA; Lightspeed VCT scanner, GE Healthcare, USA;  BrightSpeed, GE Healthcare, USA) |
| CT  tube voltage | 120 kVp | 120 kVp | 120 kVp | Spectral imaging mode switching between 120 kVp and 140 kVp |
| CT  tube current | 220 mA | 60 mA | 300 mA or 100-300 mA | 50-480mA (the duration is optimized automatically to provide similar signal strength) |
| CT  rotation time | 0.6 s | 0.6 s | 0.5 s or 0.6 s | 0.60-0.80 s |
| CT detector collimation | 64×0.625 mm | 64×0.5 mm | 16×1.2 mm or 32×1.2 mm or 64×0.625 mm | 64×0.625 mm |
| Contrast agent type | Iopamidol, Iopamiro, Bracco Sine, Shanghai, China | Iopamiro, Bracco Sine, Shanghai, China | Iopamidol, Iopamiro, Bracco Sine, Shanghai, China; iohexol, Omnipaque 300, Amersham,  Shanghai, China | Omnipaque, GE Healthcare, USA |
| Contrast agent concentration | 370 mgI/ml | 300 mgI/ml | 300 mgI/ml | 300 mgI/ml |
| Contrast agent dosage | infused 1.5 ml/kg body weight | infused 1.5 ml/kg body weight | infused 1.5 ml/kg body weight | infused 1.5 ml/kg body weight |
| Contrast agent infused rate | 3.0 ml/s | 3.0 ml/s | 3-4 ml/s | 2.0~3.3ml/s |
| Arterial phase  interval time | 35 s after injection of  contrast agent | 36 s after injection of  contrast agent | 30 s after injection of  contrast agent | 30 s after injection of  contrast agent |
| Venous phase  interval time | 70 s after injection of  contrast agent | 90 s after injection of  contrast agent | 60 s after injection of  contrast agent | 65~70 s after injection of  contrast agent |
| Image matrix | 512×512 | 512×512 | 512×512 | 512×512 |
| Field of view | 500×500 mm | 500×500 mm | 500×500 mm | 500×500 mm |
| Reconstruction image thickness | 2 mm or 5 mm | 5 mm | 1.2 mm or 2 mm or 5 mm | 5 mm |

**Reference**

1. Joensuu H. Risk stratification of patients diagnosed with gastrointestinal stromal tumor. Hum Pathol. 2008;39(10):1411-9.

2. van Griethuysen JJM, Fedorov A, Parmar C, Hosny A, Aucoin N, Narayan V, et al. Computational Radiomics System to Decode the Radiographic Phenotype. Cancer Res. 2017;77(21):e104-e7.

3. Shrout PE, Fleiss JL. Intraclass correlations: uses in assessing rater reliability. Psychological bulletin. 1979;86(2):420.

4. De Jay N, Papillon-Cavanagh S, Olsen C, El-Hachem N, Bontempi G, Haibe-Kains B. mRMRe: an R package for parallelized mRMR ensemble feature selection. Bioinformatics (Oxford, England). 2013;29(18):2365-8.

5. Friedman J, Hastie T, Tibshirani R. Regularization paths for generalized linear models via coordinate descent. Journal of statistical software. 2010;33(1):1.
